# Cancer in the Time of Coronavirus: A Call for Crisis Oncology Standards of Care

**DOI:** 10.3390/healthcare8030214

**Published:** 2020-07-17

**Authors:** Amar H. Kelkar, Christopher R. Cogle

**Affiliations:** Division of Hematology and Oncology, Department of Medicine, College of Medicine, University of Florida, Gainesville, FL 32610, USA; Christopher.Cogle@medicine.ufl.edu

**Keywords:** Coronavirus, COVID-19, cancer, crisis, disaster preparedness

## Abstract

Since the Coronavirus Disease 2019 (COVID-19) was identified as a global pandemic, health systems have been severely strained, particularly affecting vulnerable populations such as patients with cancer. In response to the COVID-19 pandemic, a variety of oncology specialty societies are making recommendations for standards of care. These diverse standards and gaps in standards can lead to inconsistent and heterogeneous care among governments, cancer centers, and even among oncologists within the same practice. These challenges highlight the need for a common nomenclature and crisis guidelines. For times of increased scarcity of resources, the National Academy of Medicine developed *Crisis Standards of Care*, defined as fairness, duty to care, duty to steward resources, transparency, consistency, proportionality, and accountability. However, we believe there is an urgent need to develop cancer-specific guidelines by convening a panel of experts from multiple specialties. These would be Crisis Oncology Standards of Care (COSCs) that are sensitive to both the individual cancer patient and to the broader health system in times of scarce resources, such as pandemic, natural disaster, or supply chain disruptions.

## 1. Introduction

In late 2019, the first cases of Severe Acute Respiratory Syndrome Coronavirus (SARS-CoV-2) of zoonotic origin and its human infection, known as Coronavirus Disease 2019 (COVID-19), were identified in the city of Wuhan, China, and spread rapidly across the world in a global pandemic [1]. This crisis has added considerable strain to already overtaxed health systems, distinctly impacting points of entry such as emergency departments and intensive care units (ICUs). While there are challenges in all fields of medicine under the public health threat of COVID-19, there are unique considerations in caring for the population of individuals with cancer given its older age, suppressed immune system (related to disease and treatment), and linkage to other risk factors such as tobacco smoking [2,3]. We believe the cancer population would benefit greatly from specific guidance on diagnostic, therapeutic, and surveillance management based on the severity of the public health crisis.

## 2. Discussion

Preliminary evidence from both China and Italy showed that cancer patients were more adversely affected by COVID-19 than standard patients [4,5]. Data in US cancer patients and an international multicenter study have also demonstrated more severe disease courses, especially after treatment with immunotherapy or surgery [4,6]. However, these data were contradicted by an 800 patient prospective cohort study by the UK Coronavirus Cancer Monitoring Project (UKCCMP) of patients with cancer and symptomatic COVID-19, which did not demonstrate increased odds of death from COVID-19 associated with anticancer therapies including cytotoxic chemotherapy, immunotherapy, hormone therapy, radiotherapy, surgery, or targeted therapy [7]. In this UKCCMP study, the strongest predictors of mortality were age, male sex, cardiovascular disease, and hypertension. Additionally, a retrospective observational study in New York demonstrated significantly higher mortality in patients with cancer and COVID-19 below the age of 50, but no significant different in patients above the age of 50 [6]. Due to the observational, imperfect nature of these datasets with numerous confounders, including notable variance between study groups, it remains difficult to clearly determine the significance of cancer and immunosuppression as risk factors for more severe disease courses of COVID-19. For the benefit of improved study quality, guidelines, and policymaking, ‘principles for collaboration in the field of cancer and COVID-19′ were outlined [8].

There is no historical precedent for management of novel checkpoint inhibitors, targeted therapies, and cellular therapies during pandemic events. However, insight can be drawn from the 2003 Severe Acute Respiratory Syndrome (SARS) outbreak in Toronto. Among the many emergency public health measures that were undertaken during that period, a key decision was to close transplant programs. Subsequently, there were concerns about the risks of resuming usual care and possible transmission of SARS from donors to recipients, and therefore a donor SARS screening tool was developed to support transplant programs. The introduction of this tool changed donor screening practices and no cases of SARS transmission from donor to recipient were or have been reported [9].

Before the COVID-19 pandemic, oncology standards of care were generally urgent, yet theoretically elective. However, guidance on cancer screening and diagnostic procedures have been considered a lower priority compared to cancer treatment. During the SARS-CoV-2 pandemic international guidelines, as well as state and local ordinances, limited “elective surgeries” inconsistently, resulting in delayed cancer diagnoses [10,11,12]. The IQVIA Institute for Human Data Science estimated that the first three months of the SARS-CoV-2 pandemic led to missing approximately 80,000 cases of cancer in the US alone [13]. Diagnostic delays may cause stage migration, where these missed cases will present with more advanced stage cancer at a later time point and will likely contribute to delayed and possibly more aggressive or expensive treatment [14].

The prevailing dogma is that cancer does not wait, so neither should our treatments. Studies have been mixed but seem to generally favor continued treatment during the pandemic. We must also consider cancer patients a vulnerable population due to the high financial and emotional cost of the underlying disease. However, these antineoplastic therapies are highly resource intensive. The intentional treatment with immunosuppressive therapies or potentially morbid surgery raises the question of whether it is ethical to increase the number of at-risk individuals when there are concerns regarding scarcity of resources, such as the availability of blood products, ICU beds, ventilators, personal protective equipment, and personnel.

A recent American Society of Clinical Oncology (ASCO) expert panel published recommendations for balancing the ethical treatment of individuals with cancer with the health of the population in the setting of resource scarcity [15]. The panel recommendations were based on the University of Pittsburgh’s *Allocation of Scarce Critical Care Resources During a Public Health Emergency* and The Hastings Center’s *Ethical Framework for Health Care Institutions Responding to Novel Coronavirus SARS-CoV-2 (COVID-19)* [16,17]. These recommendations have significant overlaps with the principles of the *Crisis Standards of Care*, defined by the National Academy of Medicine as fairness, duty to care, duty to steward resources, transparency, consistency, proportionality, and accountability [18]. There have also been several preliminary international guidelines published on the management of cancer patients in the setting of COVID-19 (Table 1) [15,19,20,21]. Additional guidelines published for specific countries and published in other languages, as well as specific disease states, have previously been summarized [22,23,24,25]. However, these triaged guidelines are mostly non-specific and may have contributed to significant inconsistency between providers and centers. This points to the larger unmet need of true Crisis Oncology Standards of Care (COSCs) that are sensitive to both the individual cancer patient and to the broader health system in times of scarce resources. The ASCO guidelines could serve as a preliminary framework for these COSCs [15].

To design COSCs, a task force of cancer control experts from multiple organizations would need to be brought together. They would first need to define common nomenclature to allow for a unified discussion, including terms like “essential” and “elective” procedures. The American Medical Association *Code of Medical Ethics* would need to be reviewed and expanded on to define triage principles and potentially specific clinical protocols in cancer care so that they can be applied fairly and consistently to all cancer patients [26]. Using the *Code*, these COSCs would need to define ethical allocation principles for both acute and chronic scarcity of personnel and product resources to avoid unequal practices between facilities. These could be expanded from the ASCO guidelines to include (1) modified sickest first (accounting for performance status and comorbidities), (2) life-years saved, and (3) modified youngest first (based on ability to tolerate oncologic therapies), while still considering utilitarian principles like maximizing number of lives saved, but avoiding first-come-first-serve allocation. Part of this ethical approach would need to prioritize urgency of treatment by histological type (e.g., acute promyelocytic leukemia or small-cell lung cancer), presence of emergent features (e.g., superior vena cava syndrome), and the resource intensity required for treatment (e.g., availability of oral chemotherapeutic options). COSCs would have to consider which clinical trials to continue and how to handle participant recruitment and management in situations of severe scarcity such as pandemics, natural disasters, and mass casualty events [27]. Oral therapies and telehealth follow-up may become more favored in these settings as trial recruitment falls [28]. One possible solution for clinical trials actively accruing patients could be to offer intermediate analysis of efficacy to justify continued study during high-risk periods; however, this could risk compromising future interpretation of these results, so this would need to be conducted thoughtfully and with input from scientific leaders and regulatory organizations. COSCs would also have to define triggers for initiation, such as interruptions to the chemotherapeutic supply chain, limited access to hospital beds or critical care services, staffing shortages or coverage by non-oncologic certified services, or inability to ensure safe management of treatment-related side effects [18]. Establishing COSCs would need to include discussion of situations in which potentially curative treatments, adjuvant therapies, or cellular therapies might be delayed and what supportive care should be used during the delay. Continuation or delay of cancer screenings needs to be discussed. Broad applications of telehealth and its reimbursement would need to be defined. Emergency approval of interstate licensure for oncology-certified clinicians for travel or telehealth may be an element of COSCs as well. 

International consensus guidelines have largely focused on ethical allocation of resources to patients with COVID-19, but do not provide specific guidance on how individual centers would prioritize diagnostics, procedures, and treatments based on urgency [15]. One exception was the interim consensus guidance prepared by the Australasian Leukemia and Lymphoma Group (ALLG) and the National Centre for Infections in Cancer that presented responses based on disease phases [29]. Another, more focused, example by Kutikov and colleagues outlined risks of cancer progression with delay of treatment [30]. Many of our patients may never develop COVID-19 but may be adversely affected by delays or rationing of essential cancer care [31]. Thus, we developed a preliminary COSC decision support tool for grading oncology treatments that could help guide health systems and oncologists in their stewardship of resources during times of crisis (Table 2). With a tool like this, the oncology community could swiftly communicate the urgency levels of oncologic care that are indicated or must be withheld as a public health threat escalates or de-escalates. Additional dimensions to consider in a decision support tool would include age, immune suppression, medication and food security, caregiver support system, and other risk factors for susceptibility in public health threats. While this COSC decision support tool was designed to address issues on a local or national level, the World Health Organization or a conglomerate of international organizations could collectively apply these recommendations internationally with targeted recommendations for individual countries if needed.

## 3. Conclusions

A mere four months ago, the leading ethical concerns were financial toxicity and disparities in access to oncology care [27]. While these issues remain, they are now complicated by a public health threat and the subsequent rationing of health care resources and delays in care. This current crisis serves as the impetus for developing guidelines for ethical rationing of resources in cancer care, but we believe that further expansion should include our COSC decision support tool and increased ethical guidance. COSCs would also need to consider prioritization by histology, chemotherapy and immunotherapy supply chain issues, scarcity of critical care resources, and clinical trials. More detailed COSC guidelines would enable the judicious use of cancer therapies, palliative care, supportive care, and end-of-life care during the current crisis and in the future.

## Figures and Tables

**Table 1:** Published Preliminary COVID-19 Cancer Guidelines and Recommendations.

Society	Recommendations	Links
ACR/ASBrS/NAPBC/NCCN/CoC *Dietz 2020*	Joint recommendations for prioritization and treatment of patients with breast cancer.	https://www.facs.org/-/media/files/quality-programs/napbc/asbrs_napbc_coc_nccn_acr_bc_covid_consortium_recommendations.ashx
ACS	General background on cancer and COVID-19, current advocacy efforts, common questions and answers, and patient information.	https://www.cancer.org/about-us/what-we-do/coronavirus-covid-19-and-cancer.html
ASCO *Marron 2020*	Ethical considerations for resource scarcity in cancer care.	https://www.asco.org/sites/new-www.asco.org/files/content-files/advocacy-and-policy/documents/JCO.20.00960.pdf?cid=DM4876&bid=41686163
ASCO	Recommendations for triage, diagnosis, treatment, infection prevention, facility management, clinical trials, and support services with links.	https://www.asco.org/sites/new-www.asco.org/files/content-files/2020-ASCO-Guide-Cancer-COVID19.pdf
ASH	Expert recommendations on management of chemotherapy, cellular therapies, and transplant for hematologic malignancies and non-malignant diseases including thrombosis.	https://www.hematology.org/covid-19
ASTRO	Recommendations for screening, treatment, and follow-up of radiation therapy during the COVID-19 pandemic.	https://www.astro.org/Daily-Practice/COVID-19-Recommendations-and-Information/Summary
EBMT	Recommendations on deferral or treatment with HSCT based on COVID-19 status of donor or recipient.	https://www.ebmt.org/sites/default/files/2020-04/EBMT-COVID-19-guidelines_v.6.1%282020-04-07%29.pdf
ESMO	Recommendations for triage during the COVID-19 pandemic by tumor type, stage, prioritization.	https://www.esmo.org/guidelines/cancer-patient-management-during-the-covid-19-pandemic
ESTRO	General recommendations on treatment and deferral with radiation therapy.	https://www.estro.org/About/Newsroom/News/Radiotherapy-in-a-time-of-crisis
NCCN *Ueda 2020*	Outline of recommendations for triage, treatment decisions, ethical considerations, and hospital management.	https://jnccn.org/view/journals/jnccn/18/4/article-p366.xml?rskey=vYDEqI&result=1
SIOP	Resource library, registry, and collaboration space for COVID-19 management in cancer.	https://global.stjude.org/en-us/global-covid-19-observatory-and-resource-center-for-childhood-cancer.html
SSO *Bartlett 2020*	Management of solid tumor surgeries and neoadjuvant/adjuvant chemotherapy including timing and deferral during the COVID-19 pandemic.	https://link.springer.com/article/10.1245/s10434-020-08461-2
WBMT	Brief recommendations regarding transplant for hematologic malignancies including potential need to delay transplants in areas with endemic or high frequency of COVID-19 infections.	https://www.wbmt.org/wp-content/uploads/2020/03/WBMT_COVID-19-2.pdf

Dietz 2020 [19], Marron 2020 [15], Ueda 2020 [20], and Bartlett 2020 [21]. ACR, American College of Radiology; ASBrS, American Society of Breast Surgeons; NAPBC, National Accreditation Program for Breast Centers; NCCN, National Comprehensive Care Network; CoC, Commission on Cancer; ACS, American Cancer Society; ASCO, American Society of Clinical Oncology; ASH, American Society of Hematology; ASTRO, American Society for Radiation Oncology; EBMT, European Society for Blood and Marrow Transplantation; ESMO, European Society for Medical Oncology; ESTRO, European Society for Radiotherapy and Oncology; SIOP, International Society of Pediatric Oncology; SSO, Society of Surgical Oncology; WBMT, Worldwide Network for Blood and Marrow Transplantation.

**Table 2:** Example Crisis Oncology Standards of Care (COSC) Decision Support Tool for Treatment Initiation.

		COSC Level			
		A	B	C	D
	Definitions:	Chronic Care	Subacute Care	Urgent Care	Emergent Care
**Modality**	**Cancer Prevention**	- Screening office visits - HPV testing - Screening chest computed tomography - Screening prostate exam and PSA testing - Routine skin examination	- Surveillance colonoscopy - High-risk skin examination - Screening telehealth visits		
	**Surgery/Procedural**	- Screening colonoscopy	- Excision of indolent mass - Presumed cancer biopsy - Surveillance telehealth visits	- Excision of malignant mass - Debulking surgery - Abscess drainage	- Threatened organ or limb - Typhlitis management - Spinal cord decompression - Fixation of orthopedic fracture
	**Radiation Therapy**	- Surveillance office visits	- Adjuvant radiation therapy - Chemoembolization - Surveillance telehealth visits	- Neoadjuvant radiation therapy - Curative radiofrequency ablation - Spinal cord or organ impingement radiation therapy - Central nervous system stereotactic radiosurgery	- Superior vena cava syndrome - Severe bony pain and symptom radiation therapy
	**Medical Oncology**	- Surveillance office visits	- Adjuvant chemotherapy - Adjuvant immuno-oncology therapy - Surveillance telehealth visits	- Superior vena cava syndrome - Visceral crisis - Neoadjuvant chemotherapy - Transfusion support	- Febrile neutropenia - Tumor lysis syndrome - Hypercalcemia of malignancy - Syndrome of inappropriate antidiuretic hormone
	**Malignant Hematology**	- Surveillance office visits	- Autologous hematopoietic cell transplantation for myeloma or lymphoma - Cellular therapies - Acute myeloid leukemia arising from antecedent hematologic disorder - Acute leukemia consolidation chemotherapy - Myeloma maintenance therapy - Myeloproliferative disease maintenance therapy - Chronic myeloid leukemia therapy - Donor lymphocyte infusions - Chronic graft-versus-host disease immune suppression - Surveillance telehealth visits	- Allogeneic hematopoietic cell transplantation - Cellular therapies - Acute leukemia and blast crisis induction and re-induction therapy - Myeloma induction and re-induction therapy - Sub-massive pulmonary embolism - Acute graft-versus-host disease immune suppression	- Acute promyelocytic leukemia - Disseminated intravascular coagulopathy - Leukostasis - Tumor lysis syndrome - Febrile neutropenia - Differentiation syndrome - Massive pulmonary embolism
	Clinical Trials	- Observational Studies	- Interventional studies with projected modest improvement in clinical outcomes - Clinical trial surveillance imaging and laboratory testing - Surveillance office visits - Surveillance telehealth visits	- Interventional studies with projected major improvement in clinical outcomes - Interventional studies for crisis-related treatments in the cancer population - Clinical trial adverse outcomes monitoring	- Interventional studies for oncologic emergencies with projected improvement in clinical outcomes
	Cancer Survivorship	- Surveillance imaging - Surveillance office visits	- Surveillance laboratory testing - Surveillance telehealth visits		

HPV, human papilloma virus; PSA, prostate-specific antigen.
